# Establishing reference values for tensiomyography-derived parameters in soccer players: insights from a systematic review, meta-analysis and meta-regression

**DOI:** 10.5114/biolsport.2025.139853

**Published:** 2024-07-31

**Authors:** Armin H. Paravlic

**Affiliations:** 1 Faculty of Sport, University of Ljubljana, Ljubljana, Slovenia; 2 Science and Research Centre Koper, Koper, Slovenia; 3 Faculty of Sports Studies, Masaryk University, Brno, Czech Republic

**Keywords:** TMG, Muscle contractile properties, Neuromuscular function, Muscle typology, Hamstrings, Football

## Abstract

This systematic literature review (SLR) aimed to comprehensively synthesize existing studies that have reported on TMG-derived parameters of lower extremities in soccer players. The PubMed, Web of Science, and EBSCOHost (including MEDLINE, SPORTDiscuss, ERIC, DOAJ, and SCOPUS) databases were searched from inception to the 31^st^ of August, 2023. Reports were eligible if they satisfied the following criteria: recruited active soccer players, with no restriction on race, sex, age, level of expertise, or health status; studies utilizing TMG for measuring muscle contractile properties. In total, 25 published journal articles from 22 original studies were included in the current review, encompassing a total of 1224 participants (4% females). The analysis considered various muscles, with the biceps femoris (BF), rectus femoris (RF), vastus lateralis (VL), vastus medialis (VM), semitendinosus (ST), gastrocnemius lateralis (GL), and gastrocnemius medialis (GM) being investigated. Significant variations were observed in TMG parameters across different muscles, age categories, and levels of play. The quality of evidence varied from low to moderate for all analyses. The meta-regression analysis indicated that age moderated several TMG-derived parameters in lower limb muscles including BF Vc, RF Td and Vc, ST Dm and sustain time, VL Dm, Tc, Td and relaxation time (Tr), and VM Tc, Td and Tr, respectively. In conclusion, the current review illuminated the multifaceted applications of TMG in assessing lower extremity muscles in soccer players. Beyond evaluating muscle contractile properties in various superficial muscles of the lower limbs in soccer players, TMG-derived parameters may serve as potentially valuable markers in identifying neuromuscular risk factors for anterior cruciate ligament injuries and predicting hamstring-related injuries.

## INTRODUCTION

Neuromuscular function is regularly assessed in sports settings, with sports scientists and strength and conditioning coaches utilizing these measures to strategically plan and enhance training programs for improved sports performance and injury prevention. Over the past two decades, tensiomyography (TMG) has emerged as a promising tool for precisely measuring neuromuscular function [1, 2]. TMG is a relatively recent method that assesses the contractile properties of superficial skeletal muscles by examining lateral muscle deformation induced by single electrical stimuli [1]. In comparison to other measurement methods considered the gold standard in evaluating neuromuscular function, such as isokinetic dynamometry and force plates, TMG offers an advantage by being independent of an athlete’s motivation or volitional effort, both of which significantly influence athletic performance [3, 4].

TMG has been extensively utilized for measuring muscle adaptations across various settings [2, 5–8]. While TMG response provides several time and distance-related parameters of muscle contraction, the time of contraction (Tc) and maximal displacement amplitude i.e., displacement measure (Dm), have consistently proven to be the most reliable parameters [9–11] and hold clinical relevance [5, 7, 12, 13]. For example, reduced Tc values suggest a muscle with a prevalence of fast-twitch muscle fibres [14, 15]. On the other side, Dm offers insights into muscle structure, with increased Dm correlating well with decreased muscle stiffness following the 35-days of bed rest [5]. Moreover, alterations in Dm and the half-relaxation time (Tr) have demonstrated to be the most sensitive measures of muscle fatigue [16, 17], with higher values indicating fatigued state [17]. In case of pathology, such as anterior cruciate ligament (ACL) injury, increased Tr values may signify muscles that are less resistant to fatigue [18]. Consequently, TMG stands as a reliable tool for non-invasive estimation of predominant skeletal muscle fibres [12, 15], monitoring muscle fatigue [17, 19], evaluating training and rehabilitation-induced adaptations [6, 7, 20–22] and lastly, assessing neuromuscular risk factors associated with ACL injuries [18, 23] and hamstring injuries [13].

To date, numerous studies have employed TMG in the investigation of soccer players’ neuromuscular characteristics [24–33]. A subset of these studies focused on assessing bilateral asymmetry in elite and sub-elite male futsal players [34] and soccer players in general [26, 35, 36]. For instance, Gill et al. [26] found no significant difference in TMG parameters between dominant and non-dominant legs among Brazilian elite soccer players. Similarly, with the exception of vastus medialis (VM) Tc, rectus femoris (RF) Tr and sustain time (Ts), and biceps femoris (BF) Ts, Alvarez-Diaz [36] reported non-significant difference in the majority of TMG variables evaluated in injury-free, competitive Spanish soccer players. Consequently, the argument can be made that leg dominance in male soccer players, without a history of musculoskeletal injuries does not exert a significant influence on TMG-derived parameters [4, 26, 34–36].

Conversely, TMG has proven to be a valuable tool for evaluating neuromuscular risk factors associated with ACL injuries [18]. In this context, the authors conducted a comparative analysis of lower extremity TMG parameters between the healthy side of soccer players with ACL injuries and those of a gender-and sport-matched healthy control group [18]. The findings revealed that time-related parameters in vastus lateralis (VL) and RF muscles, such as Tr, were 71% and 61% higher in ACL-injured players compared to controls. Additionally, RF exhibited 7% and 31% higher Tc and Ts in ACL-injured players compared to controls, respectively. Lastly, the Dm of the BF was found to be 48% higher in ACL-injured players. Later results suggest that fatigue resistance and muscle stiffness of the hamstring muscles may be significant risk factors for sustaining an ACL injury [18].

While numerous studies have employed TMG in assessing soccer players [24–33], there remains a gap in understanding the absolute reference values for key TMG parameters related to lower extremity muscles in this population. With the increasing adoption of TMG among soccer professionals, establishing these reference values becomes crucial for potential talent identification [37], guiding optimal strength and conditioning practices [20, 38] and rehabilitation planning [36, 39]. Having these benchmarks available would enhance the effectiveness of using TMG in the soccer domain, providing valuable insights for practitioners in tailoring interventions to meet the specific neuromuscular needs of soccer players.

The main objective of this systematic literature review (SLR) is to comprehensively summarize existing studies that have reported on TMG-derived parameters of lower extremities in soccer players. Furthermore, this study aimed to furnish reference values, both in a general context and with consideration to different muscles, players’ age, level of play, sex, leg dominance and playing position, respectively.

## MATERIALS AND METHODS

The protocol for current SLR with meta-analysis was performed by the Preferred Reporting Items for Systematic Reviews and Meta-Analyses (PRISMA) guidelines [40]. The study protocol was prospectively registered at the Open Science Framework online registry: ID: osf.io/nm3wb [41].

### Search Strategy

The PubMed, Web of Science, and EBSCOHost (including MEDLINE, SPORTDiscuss, ERIC, DOAJ and SCOPUS) databases were searched from inception to the 31^st^ of August, 2023. No restriction on the year of publication or language was applied. In all databases, the following keywords or phrases were used: “Tensiomyography”, “soccer” and “football”. The Boolean “OR” and “AND” were used where possible. For example PubMed database was searched with the following string: (tensiomyography [Title/Abstract]) AND ((soccer [Title/Abstract]) OR (football [Title/Abstract])). The Web of Science was searched as follows: (tensiomyography OR TMG) AND (soccer OR football), while EBSCOhost was searched by using following words and Booleans: tensiomyography OR TMG AND (football or soccer). Additionally, on the 1^st^ of September 2023, the author conducted a thorough search of the TMG-BMC website [42] to find additional reports in case any reports were not identified through primary databases search. An additional search of TMG-BMC was conducted to enhance the comprehensiveness of a literature search, as it contains unique literature particularly related to TMG.

### Inclusion and Exclusion Criteria

The inclusion criteria were formulated based on the PICOS approach [40]. Reports were eligible if they satisfied the following criteria:

– Population (P) – recruited active soccer players, with no restriction on race, sex, age, level of expertise, or health status;
– Intervention (I) – No interventions were considered in this review.

In case of interventional studies, the baseline values off all groups were considered;

– Comparison (C) – level of play (Participant Classification Frame-work – Tier 1–5) [43], and age (U14, U16, U19, U21, Seniors);
– Outcome (O) – Contractile muscle properties measured by TMG. The following TMG-derived parameters were included: a) Time delay (Td), Tc, Ts, Tr, Dm, and Velocity of contraction (Vc);
– Study Design (S) – no restrictions were placed to study design.

### Screening Strategy

The screening of the online databases and the reports was performed by the author (AP). After the first screening by AP, the screening was performed by researcher (KD), and based on a compromise between them, the study was included or excluded from the review.

### Data extraction

Information pertaining to the study design, population characteristics (sample size, level of play, Tier classification, age, sex, body height, body weight, body mass index [BMI]), methodological consideration of TMG assessment (leg dominance determination protocol, muscle measured, reported parameters, part of the season when the measurements were conducted) were systematically extracted from the original studies that have been included in the present review (Table 1 and Table 2). The extraction process employed aims to provide a comprehensive understanding of the methodologies used across diverse studies, facilitating a thorough analysis and synthesis of the collective evidence.

**TABLE 1:** Systematic overview of the included studies in the systematic review and meta-analysis with their characteristics and relevant outcomes (Part I)

Study	Country	Study design	Sample size	Expertise level	Experience (years)	Teir Classification / Level of play	Age categories	Health status	Sex
Alentorn-Geli et al. 2015 [87]	Spain	CSS	78	Competitive	NR	Tier 3 – Highly Trained	Senior	Healthy vs. ACL tear	Male

Alentorn-Geli et al. 2015 [88]	Spain	CSS	78	Competitive	NR	Tier 3 – Highly Trained	Senior	Healthy vs. ACL tear	Male

Alvarez-Diaz et al. 2014 [36]	Spain	CSS	38	Competitive	NR	Tier 3 – Highly Trained	Senior	Healthy	Male

Alvarez-Diaz et al. 2016 [89]	Spain	CSS	78	Competitive	NR	Tier 3 – Highly Trained	Senior	Healthy vs. ACL tear	Male

Alvarez-Diaz et al. 2016 [90]	Spain	CSS	40	Competitive	NR	Tier 3 – Highly Trained	Senior	Injured	Male

Beato et al. 2021 [31]	UK	EXP	31	Amateur	NR	Tier 2 – Trained/Developmental	Senior	Healthy	Male

Calderón-Pellegrino et al. 2020 [91]	Spain	EXP	32	Spanish 3^rd^ division	NR	Tier 2 – Trained/Developmental	Senior	Healthy	Male

Fernández-Baeza et al. 2022 [92]	Spain	CSS	27	Professional	15 +	Tier 4 – Elite	Senior	Healthy	Male

García-García et al. 2016 [93]	Spain	CSS	21	Professional	NR	Tier 4 – Elite	Senior	Healthy	Male

García-García et al. 2017 [94]	Spain	CSS	16	Professional	NR	Tier 4 – Elite	Senior	Healthy	Male

García-Manso et al. 2011 [95]	Spain	EXP	12	Spanish 2^nd^ division	NR	Tier 3 – Highly Trained	Senior	Healthy	Male

Gil et al. 2015 [96]	Brazil	CSS	20	Elite	NR	Tier 4 – Elite	Senior	Healthy	Male

López-Fernández et al. 2018 [97]	Spain	EXP	16	Amateur	NR	Tier 2 – Trained/Developmental	Senior	Healthy	Male

Loturco et al. 2016 [71]	Brazil	EXP	22	Elite	NR	Tier 4 – Elite	Senior	Healthy	Male

Loturco et al. 2018 [98]	Brazil	CSS	24	Elite	NR	Tier 4 – Elite	Senior	Healthy	Male

Padrón-Cabo et al. 2023 [33]	Spain	CSS	121	Elite	4 to 8	Tier 3 – Highly Trained	U16 (13 to 15)	Healthy	Male

Pajović et al. 2023 [32]	Serbia	CSS	57	Elite	5 +	Tier 4 – Elite	Senior	Healthy	Male

Paravlic et al. 2022 [51]	Slovenia	CSS	52	Elite	10.9 ± 3.5	Tier 4 – Elite	U21 (19 to 21)	Healthy	FeMale

Paravlic et al. 2022 [53]	Slovenia	CSS	266	Elite	10.4 ± 3.5	Tier 4 – Elite	Senior	Healthy	Male

Redd et al. 2 021 [99]	USA	EXP	15	Collegiate	NR	Tier 3 – Highly Trained	U21 (19 to 21)	Healthy	Male

Rey et al. 2012 [29]	Spain	CSS	78	Spanish 1^st^ division	4 to 15	Tier 4 – Elite	Senior	Healthy	Male

Rey et al. 2012 [30]	Spain	EXP	31	Professional	NR	Tier 3 – Highly Trained	Senior	Healthy	Male

Rey et al. 2020 [100]	Spain	EXP	19	Professional	4 to 10	Tier 4 – Elite	Senior	Healthy	Male

Rusu et al. 2013 [101]	Romania	EXP	30	Junior	NR	Tier 2 – Trained/Developmental	U19 (16 to 18)	Healthy	Male

Sánchez-Sánchez et al. 2019 [62]	Spain	EXP	62	Elite	NR	Tier 3 – Highly Trained	U16 (13 to 15)	Healthy	Male

CSS – cross sectional study; EXP – experimental study; NR – not reported; ACL – anterior cruciate ligament.

**TABLE 2:** Systematic overview of the included studies in the systematic review and meta-analysis with their characteristics and relevant outcomes (Part II)

Study	Age	Body Height	Body Mass	Measured leg	Leg dominance determination protocol	Muscles measured	Reported parameters	Part of the season
Alentorn-Geli et al. 2015 [87]	21.7 ± 5.85	175 ± 10	71.6 ± 8.85	Both	NR	VM, VL, RF, ST, BF	Dm, Td, Tc, Ts, Tr	NR

Alentorn-Geli et al. 2015 [88]	21.7 ± 5.85	175 ± 10	71.6 ± 8.85	Both	NR	VM, VL, RF, ST, BF	Dm, Td, Tc, Ts, Tr	NR

Alvarez-Diaz et al. 2014 [36]	21.1 ± 4.9	175 ± 7	71.5 ± 10	Both	NR	VM, VL, RF, ST, BF, GM, GL	Dm, Td, Tc, Ts, Tr	NR

Alvarez-Diaz et al. 2016 [89]	21.7 ± 5.85	175 ± 10	71.6 ± 8.85	Both	NR	VM, VL, RF, ST, BF	Dm, Td, Tc, Ts, Tr	NR

Alvarez-Diaz et al. 2016 [90]	22.3 ± 6.9	175 ± 10	71.7 ± 7.7	Both	NR	VM, VL, RF, ST, BF, GM, GL	Dm, Td, Tc, Ts, Tr	NR

Beato et al. 2021 [31]	21 ± 4	182 ± 4	77.0 ± 5.2	Dominant	NR	VM, VL, RF	Dm, Tc, Td	NR

Calderón-Pellegrino et al. 2020 [91]	23 ± 5	177 ± 6	71.2 ± 6.7	Both	NR	BF and RF	Td, Tc, Dm	Off-season

Fernández-Baeza et al. 2022 [92]	25.04 ± 4.5	179 ± 6	75.5 ± 7.7	Both	NR	BF and ST	Tc, Dm	Pre-season (preparatory period)

García-García et al. 2016 [93]	27.2 ± 3.3	180.2 ± 4.8	74.2 ± 5.6	Both	NR	VM, VL, RF, BF	Tc, Dm, Td, Ts, Tr	In season (competition)

García-García et al. 2017 [94]	28.2 ± 4.4	178.8 ± 6.0	74.5 ± 4.3	Both	NR	VM, VL, RF, BF	Dm, Td, Tc, Ts, Tr, Vc, LS, FS right, FS left	Pre-season (preparatory period)

García-Manso et al. 2011 [95]	25.89 ± 5.86	176.86 ± 6.49	73.46 ± 6.38	Dominant	NR	VL	Dm, Td, Tc, Ts, Tr, Vc,	NR

Gil et al. 2015 [96]	23.3 ± 4.8	183.5 ± 6.6	77.8 ± 7.5	Both	NR	BF and RF	Dm, Tc	NR

López-Fernández et al. 2018 [97]	22.17 ± 3.43	177.12 ± 5.24	74.42 ± 4.87	Both	NR	BF and RF	Tc, Td, Tr, Dm, Ts	NR

Loturco et al. 2016 [71]	23.8 ± 4.2	177 ± 7	76.2 ± 8	Dominant	NR	BF and RF	Tc, Td, Dm, Vc	Pre-season (preparatory period)

Loturco et al. 2018 [98]	23.9 ± 4.6	179.1 ± 9.2	77.9 ± 10.4	Both	Ball-kicking leg	BF and RF	Tc, Td, Dm, Vc	Pre-season (preparatory period)

Padrón-Cabo et al. 2023 [33]	14.98 ± 1.83	167.38 ± 10.37	60.65 ± 11.69	Dominant	NR	BF and RF	Dm, Tc, Td, Vc	In season (competition)

Pajović et al. 2023 [32]	25.46 ± 4.83	184.03 ± 5.77	80.25 ± 6.49	Both	NR	RF, VM, VL, BF, ST	Tc, Ts, Tr, Dm, Td	Off-season

Paravlic et al. 2022 [51]	18.33 ± 1.77	167.87 ± 6.33	60.13 ± 6.12	Both	Ball-kicking leg	BF, RF, VL, VM, GL,	GM, TA Td, Tc, Ts, Tr, Dm	Pre-season (preparatory period)

Paravlic et al. 2022 [53]	24.97 ± 4.92	181.53 ± 6.46	76.19 ± 8.46	Dominant	Ball-kicking leg	BF, VL, VM	Td, Tc, Ts, Tr, Dm	Pre-season (preparatory period)

Redd et al. 2021 [99]	20.25 ± 1.01	177.23 ± 6.64	79.55 ± 6.0	Both	NR	BF and RF	Td, Tc, Dm	In season (competition)

Rey et al. 2012 [29]	26.4 ± 4.4	179.2 ± 5.3	75.8 ± 5.3	Dominant	NR	BF	Dm, Td, Tc, Ts, Tr	In season (competition)

Rey et al. 2012 [30]	23.5 ± 3.4	179.9 ± 5.1	75.7 ± 4.2	Dominant	NR	BF and RF	Dm, Td, Tc	In season (competition)

Rey et al. 2020 [100]	26.0 ± 4.1	180.2 ± 4.2	77.5 ± 3.5	Dominant	NR	BF and RF	Dm, Tc, Vc	In season (competition)

Rusu et al. 2013 [101]	16	170	52	Both	NR	RF	Dm, Tc	Pre-season (preparatory period)

Sánchez-Sánchez et al. 2019 [62]	14.63 ± 2.0	167 ± 10.5	58.75 ± 12.52	Dominant	NR	BF and RF	Td, Tc, Dm	In season (competition)

NR – not reported; BF – Biceps Femoris; ST – Semitendinosus; VL – Vastus Lateralis; VM – Vastus Medialis; RF – Rectus Femoris; GL - Gastrocnemius Lateralis; GM – Gastrocnemius Medialis; TA – Tibialis Anterior; Dm – Displacement measure; Tc – Contraction time; Td – Delay time; Tr – Relaxation time; Ts – Sustain time; Vc – Velocity of contraction; LS – Lateral symmetry; FS – Functional symmetry

### Quality of evidence assessment

The Quality Assessment Tool for Observational Cohort and Cross-Sectional Studies was used to assess the methodological strength and risk of bias of the 25 studies included in the review [44]. The methodological assessment of the studies was performed independently by AP and KD. The National Heart, Lung, and Blood Institute (NHL-BI) quality assessment tool consists of a checklist of 14 questions designed to assess the internal validity (potential risk of selection, information, or measurement bias) of cross-sectional and cohort studies. The criteria were answered ‘yes’, ‘no’, or other (not specified; not applicable; not reported). The total score would be the number of affirmative responses. For the qualitative assessment of the final score, scores higher than 10 were considered good, those lower than 5 were considered weak, and those falling in the range 5 to 9 represented moderate-quality studies. All included studies were rated as good, fair, or poor quality on the basis of a rating sheet with quality assessment instructions. Furthermore, The Grading of Recommendations, Assessment, Development, and Evaluations (GRADE) was used to evaluate the quality of evidence for each study included in the present meta-analysis. For each potential risk factor, including design limitations (NHLBI score of included studies < 6), imprecision (less than 300 participants for each pooled outcome measure), and inconsistency (moderate to high heterogeneity; I^2^ ≥ 50%), the level of certainty was adopted as high, moderate, low and very low [45].

### Statistical analysis

Statistical analysis was performed with SPSS statistical software (version 29.0, IBM Inc, Chicago, United States of America). Due to large heterogeneity observed for each meta-analysis, data were analysed using a random effect model. The method of restricted maximum likelihood (REML) with Knap-Hartung standard error adjustment was used for all analyses [46]. The REML method for all analyses was chosen, as it provides less biased estimates of heterogeneity variance compared to other methods, especially in meta-analyses combining small and large studies [46]. Egger’s test was performed on collected data to provide statistical evidence of publication bias. Given the only TMG was used to analyse muscle contractile properties and the variability of the methods and units reported did not exist, a mean pooled values (Mean) along with 95% confidence intervals (CI) were calculated for all outcome measures. Heterogeneity was assessed with I^2^ statistic that indicates the percentage of variability across studies due to heterogeneity rather than chance. Values of 25%, 50% and 75% represent low, moderate and high heterogeneity [47]. Moreover, to investigate the potential moderators on variables of interest, several subgroup meta-analyses were performed by comparing: a) Level of play (Tier 2 / Trained vs. Tier 3 / highly trained vs. Tier 4 / Elite and; b) age categories (U14 vs. U16 vs. U19 vs. U21 vs. Seniors). The data necessary for categorical variables were directly extracted from the original reports. For example, the level of play was derived based on the data provided within a report and categorized as recommended [43], while the average age of participants was used to categorize a players to different age groups. Additionally, random-effects meta-regression using the REML was performed to examine whether the age of soccer players influence the TMG-derived parameters. To minimize the risk of overfitting, a meta-regression was performed when a minimum of 10 studies were eligible per examined covariate [48]. A level of p < 0.05 was adopted as statistically significant. In addition, a GraphPad Prism (version 8.0) and Microsoft Office PowerPoint were used for a graphical presentation of the data.

## RESULTS

**FIG. 1:**
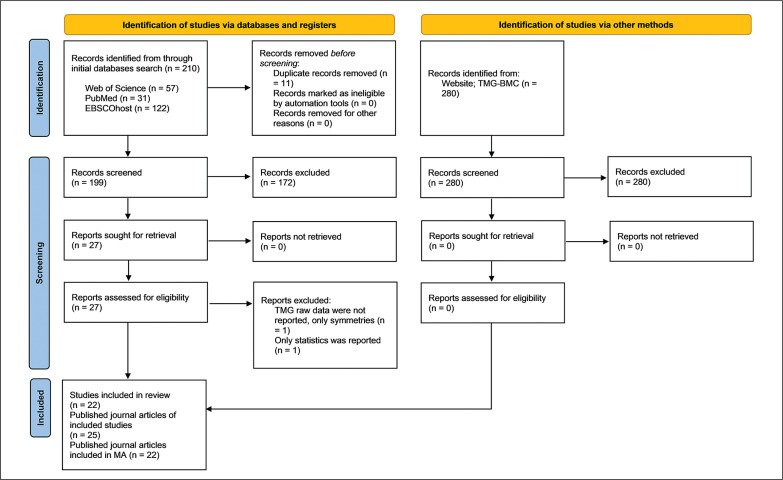
PRISMA flow diagram of study selection process.

In the initial search across databases, a total of 210 reports were identified, with an additional 280 reports found on the TMG BMC website (Figure 1). After removing duplicates, 199 reports remained. Following title and abstract screening, 172 records were excluded from the primary online database search. Additionally, all reports found through TMG-BMC were excluded: 81 were deemed ineligible, and 199 were duplicates of reports identified through other databases. The full texts of 27 reports were assessed for eligibility. Each report underwent a thorough review, and key study characteristics, including author, year of publication, country of the main author, original study design, aim of the original study, participants’ information (sample size, level of play, playing experience, Tier classification, age of players, sex, body height, body mass, BMI, leg dominance, and measured leg), as well as TMG-derived parameters (Td, Tc, Tr, Ts, Dm, and Vc), were extracted and presented in Table 1 and Table 2.

Two studies were excluded; one did not report raw TMG data, only symmetries [49], and another reported only statistics without including TMG-derived parameters [50]. Consequently, 25 journal articles from 22 original studies were included in this SLR, encompassing a total of 1224 participants (with only 4% being female soccer players, noted in one study [51]) (Fig. 1). The mean age of players was 23.0 ± 3.6 years (range 12.6 – 28.2). The minimum number of players, 12, was recruited in the study of García-Manso et al. [52], while Paravlic et al. [53] recruited the highest number of soccer players (N = 266). The included studies comprised seven experimental studies and 15 cross-sectional studies.

Among the investigated muscles, the BF was the most frequently studied (22 reports), followed by the RF (21 reports), VL (12 reports), VM (11 reports), semitendinosus (ST) (seven reports), and gastrocnemius lateralis (GL) and gastrocnemius medialis (GM) (both three reports). All studies reported parameters Dm and Tc, while Td, Tr, Ts, and Vc were of less interest in the literature.

Due to lack of data reported in original studies, meta-analysis investigating moderating effect of players’ sex, leg dominance, injured vs. non-injured leg and playing position was not conducted. Thus, data were analysed for soccer players who were assessed free of musculoskeletal injuries.

### Quality of evidence

The median NHLBI score was 6.3 ± 1.7, with values ranging from 4 to 10, suggesting that the included studies were generally of fair quality (Supplementary table 1). The quality of evidence for all analyses varied from low to moderate quality (Table 3).

**TABLE 3:** Grading of Recommendation, Assessment, Development and Evaluations (GRADE) for the overall results summarized

									Reasons for downgrade		

Parameter	Outcome	Participants (n)	Mean	LLCI	HLCI	I^2^	NHLBI score	GRADE score	NHBI less than 6	< 300 participants	I^2^ 50%
Dm	BF	1071	5.26	4.66	5.86	96.4	6.6	Moderate quality	No	No	Yes
	GL	114	3.73	3.59	3.88	0.0	5.0	Low quality	Yes	Yes	No
	GM	114	3.07	2.92	3.21	0.0	5.0	Low quality	Yes	Yes	No
	RF	842	8.89	8.37	9.42	93.4	6.2	Moderate quality	No	No	Yes
	ST	282	7.27	5.97	8.57	92.8	6.8	Low quality	No	Yes	Yes
	VL	591	5.32	4.72	5.93	93.8	6.0	Moderate quality	No	No	Yes
	VM	579	7.36	6.75	7.97	95.8	6.0	Moderate quality	No	No	Yes

Tc	BF	1071	28.74	26.79	30.68	96.9	6.6	Moderate quality	No	No	Yes
	GL	114	20.88	20.02	21.75	0.0	5.0	Low quality	Yes	Yes	No
	GM	114	22.09	21.47	22.70	0.0	5.0	Low quality	Yes	Yes	No
	RF	842	29.32	27.95	30.69	97.1	6.2	Moderate quality	No	No	Yes
	ST	282	41.44	38.27	44.62	87.4	6.8	Low quality	No	Yes	Yes
	VL	591	24.56	22.82	26.31	99.1	6.0	Moderate quality	No	No	Yes
	VM	579	25.34	23.73	26.94	99.2	6.3	Moderate quality	No	No	Yes

Td	BF	958	23.44	22.88	24.00	95.0	6.4	Moderate quality	No	No	Yes
	GL	114	18.36	18.21	18.51	0.0	5.0	Low quality	Yes	Yes	No
	GM	114	20.00	19.03	20.97	27.0	5.0	Low quality	Yes	Yes	No
	RF	723	25.15	24.70	25.60	85.5	6.3	Moderate quality	No	No	Yes
	ST	228	24.33	23.84	24.83	0.1	6.0	Moderate quality	No	Yes	No
	VL	591	23.07	22.30	23.83	97.8	6.0	Moderate quality	No	No	Yes
	VM	579	21.83	21.42	22.25	94.1	6.3	Moderate quality	No	No	Yes

Tr	BF	627	57.62	47.37	67.88	98.3	6.5	Moderate quality	No	No	Yes
	GL	114	44.03	36.48	51.57	0.0	5.0	Low quality	Yes	Yes	No
	GM	114	53.20	48.86	57.54	0.0	5.0	Low quality	Yes	Yes	No
	RF	361	70.18	59.98	80.38	89.1	6.1	Moderate quality	No	No	Yes
	ST	228	63.53	50.06	77.00	92.1	6.0	Low quality	No	Yes	Yes
	VL	560	47.93	39.19	56.68	93.5	6.3	Moderate quality	No	No	Yes
	VM	548	65.15	50.40	79.91	99.1	6.7	Moderate quality	No	No	Yes

Ts	BF	627	193.20	174.12	212.28	96.2	6.5	Moderate quality	No	No	Yes
	GL	114	196.11	189.31	202.91	0.0	5.0	Low quality	Yes	Yes	No
	GM	114	181.89	173.42	190.37	0.0	5.0	Low quality	Yes	Yes	No
	RF	361	115.36	102.46	128.27	97.6	6.1	Moderate quality	No	No	Yes
	ST	228	155.66	145.94	165.38	70.4	6.0	Low quality	No	Yes	Yes
	VL	560	89.61	74.73	104.49	94.9	6.3	Moderate quality	No	No	Yes
	VM	548	186.17	175.13	197.20	96.9	6.7	Moderate quality	No	No	Yes

Vc	BF	243	0.13	0.10	0.15	95.8	7.0	Low quality	No	Yes	Yes
	RF	243	0.64	-0.37	1.65	100.0	7.0	Low quality	No	Yes	Yes
	VL	45	0.22	0.20	0.24	77.4	6.0	Low quality	No	Yes	Yes
	VM	16	0.29	0.00	0.00	0.0	7.0	Moderate quality	No	Yes	No

BF – Biceps Femoris; ST – Semitendinosus; VL – Vastus Lateralis; VM – Vastus Medialis; RF – Rectus Femoris; GL – Gastrocnemius Lateralis; GM – Gastrocnemius Medialis; Dm – Displacement measure; Tc – Contraction time; Td – Delay time; Tr – Relaxation time; Ts – Sustain time; Vc – Velocity of contraction; I^2^ – Heterogeneity; NHLBI – National Heart, Lung, and Blood Institute study quality assessment tool; Yes – the criterion is satisfied to downgrade the quality of evidence.; No – the criterion is not satisfied to downgrade the quality of evidence.

### TMG-derived parameters in general Time of contraction (Tc)

**FIG. 2:**
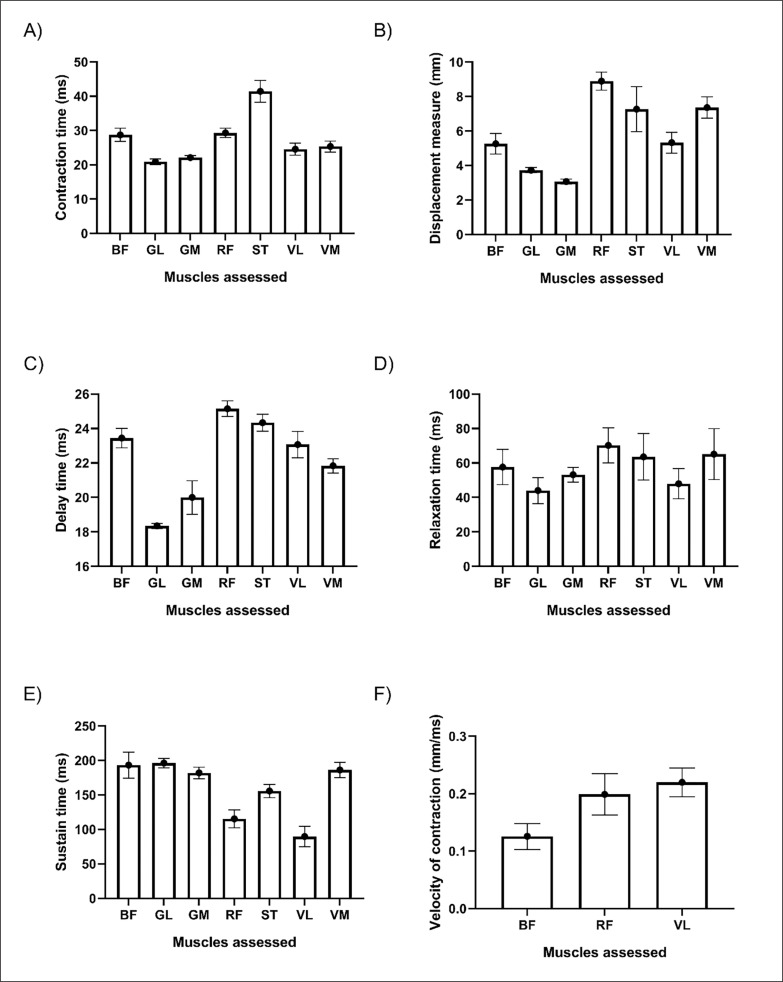
Comparison of TMG-derived variables between different lower limb muscles in soccer player.

The pooled mean value of Tc for all assessed muscles is presented in Fig. 2, A. Results showed that shortest Tc was found in GL muscle (20.88 ms), followed by GM (22.09 ms), VL (24.56 ms), VM (25.34 ms), RF (29.32 ms), BF (28.74 ms) and ST (41.44 ms), respectively. Subgroup analysis demonstrated that these differences were significant between muscles (Q = 351.24, p < 0.001).

### Displacement measure (Dm)

The pooled mean value of Dm for all assessed muscles is presented in Fig. 2, B. Results showed that the shortest Dm was found in GM muscle (3.07 mm), followed by GL (3.73 mm), BF (5.26 mm), VL (5.32), ST (7.27 mm), VM (7.36 mm) and RF (8.89 mm), respectively. Subgroup analysis demonstrated that these differences were significant between muscles (Q = 663.29, p < 0.001).

### Time delay (Td)

The pooled mean value of Td for all assessed muscles is presented in Fig. 2, C. Results showed that shortest Td was found in GL muscle (18.36 ms), followed by GM (20.00 ms), VM (21.83 ms), VL (23.07 ms), BF (23.44 ms), ST (24.33 ms) and RF (25.15 ms), respectively. Subgroup analysis demonstrated that these differences were significant between muscles (Q = 1115.35, p < 0.001).

### Relaxation time (Tr)

The pooled mean value of Tr for all assessed muscles is presented in Fig. 2, D. Results showed that the shortest Tr was found in GL muscle (44.03 ms), followed by VL (47.93 ms), GM (53.20 ms), BF (57.62 ms), ST (63.53 ms), VM (65.15 ms) and RF (70.18 ms), respectively. Subgroup analysis demonstrated that these differences were significant between muscles (Q = 33.07, p < 0.001).

### Sustain time (Ts)

The pooled mean value of Ts for all assessed muscles is presented in Fig. 2, E. Results showed that the shortest Tr was found in VL muscle (89.61 ms), followed by RF (115.36 ms), ST (155.66 ms), GM (181.89 ms), VM (186.17 ms), BF (193.20 ms) and GL (196.11 ms), respectively. Subgroup analysis demonstrated that these differences were significant between muscles (Q = 283.113, p < 0.001).

### Contraction velocity (Vc)

The pooled mean value of Vc for all assessed muscles is presented in Fig. 2, F. Results showed that the greatest Vc was found in VL muscle (0.220 mm/ms) followed by RF (0.199 mm/ms), and BF (0.126 mm/ms), respectively. Subgroup analysis demonstrated that these differences were significant between muscles (Q = 37.208, p < 0.001).

### Comparison of TMG-derived parameters between age categories and level of play

A tabular presentation comparing TMG-derived parameters across different age categories and levels of play can be found in Supplementary Tables 2 and 3, respectively.

### Biceps femoris muscle

TMG-derived parameters of the BF exhibited significant variation across age categories (Dm, Q = 72.75, p < 0.001; Tc, Q = 70.02, p < 0.001; Td, Q = 12.46, p = 0.014;). The smallest Dm amplitude was found in the U21 (3.15 mm), followed by Senior players (5.18 mm), U14 (5.98 mm), U19 (6.12 mm), and U16 (6.25 mm), respectively. Tc was the shortest in the U21 category (22.17 ms), followed by Senior players (27.92), U16 (32.83 ms), U19 (34.60 ms), and U14 (36.62 ms), respectively. Lastly, Td values were shortest in the U21 category (21.93 ms), followed by Senior players (23.33 ms), U16 (24.03 ms), U19 (24.14 ms) and U14 (24.68 ms), respectively.

Considering the level of play, significant differences were observed in Dm (Q = 31.13, p < 0.001), Tc (Q = 33.77, p < 0.001), Td (Q = 11.04, p = 0.004), and Vc (Q = 6.96, p = 0.008) between groups. Dm was found to be the shortest in Elite players (4.97 mm), followed by Highly trained players (5.25 mm) and Trained players (7.71 mm). A similar trend was observed for Tc, where Elite players exhibited the shortest time (27.23 ms), followed by Highly trained (29.39 ms) and Trained players (41.86). Td was shortest in Highly trained players (23.27 ms), followed by Elite (23.39 ms) and Trained players (24.55 ms). Vc was greater in Elite players compared to Highly trained players (0.13 mm/ms vs. 0.10 mm/ms).

### Rectus femoris muscle

For the RF muscle, subgroup analysis was conducted considering age-categories for Dm only, revealing significant variation between age-categories (Q = 13.35, p = 0.010). Senior players showed a greater Dm amplitude compared to U19 players (9.07 mm vs. 7.95 mm).

Considering the level of play, significant differences were observed in Dm (trend Q = 4.67, p = 0.097), Tc (Q = 6.98, p = 0.030), Tr (Q = 12.69, p = 0.002), Ts (Q = 8.51, p = 0.014), and Vc (Q = 5.18, p = 0.023) between groups. Dm was found to be the shortest in Trained players (8.46 mm), followed by Elite players (8.77 mm), and Highly trained (9.50 mm), respectively. A similar pattern was observed for Tc, where Trained players showed the shortest contraction time (27.00 ms), followed by Elite players (28.60 ms), and Highly trained players (32.33). Tr was shortest in Highly trained players (46.72 ms), followed by Elite (73.60 ms), and Trained players (95.23 ms). Ts was highest in Trained players (145.85 ms), followed by Elite players (119.23 ms), and Highly trained players (88.26 ms). Lastly, Vc was greater in Elite players compared to Highly trained players (0.21 mm/ms vs. 0.16 mm/ms).

### Vastus lateralis muscle

For the VL muscle, subgroup analysis based on age categories was not feasible as only data for senior players were reported.

Considering the level of play, significant differences were observed in Dm (Q = 13.34, p < 001), Tc (Q = 42.29, p < 0.001), Td (Q = 11.19, p = 0.004), Tr (Q = 5.85, p = 0.016), and Ts (trend – Q = 3.32, p = 0.068) between groups. For Vc, there were insufficient data to conduct a subgroup analysis. Dm was found to be the shortest in Trained players (4.29 mm), followed by Elite players (5.47 mm), and Highly trained players (5.72 mm). A similar pattern was observed for Tc, where Trained players exhibited the shortest contraction time (19.77 ms), followed by Elite players (24.94 ms) and Highly trained players (26.14). Td was shortest in Trained players (21.47 ms), followed by Highly trained players (22.66 ms), and Elite players (23.60 ms). Tr was shorter in Highly trained players then in Elite players (31.99 ms vs 55.47 ms). A parallel observation was found for Ts, indicating a lower Ts time in Highly trained players compared to Elite players (69.05 ms vs 98.68 ms).

### Vastus medialis muscle

For the VM muscle, subgroup analysis based on age categories was not feasible as only data for senior players were reported.

Considering the level of play, significant differences were observed in Dm (Q = 7.63, p = 0.022), Tc (Q = 31.94, p < 0.001), Td (Q = 62.46, p = 0.004), and Ts (trend – Q = 8.13, p = 0.004) between groups. For Vc, there were insufficient data to conduct a sub-group analysis. Dm was found to be the shortest in Trained players (7.02 mm), followed by Elite players (7.26 mm), and Highly trained players (7.94 mm). A nearly parallel pattern was observed for Tc, where Trained players exhibited the shortest contraction time (21.36 ms), followed by Highly trained players (25.60 ms) and Elite players (25.70 ms). Td was shortest in Highly trained players (20.66 ms), followed by Trained players (21.42 ms), and Elite players (22.29 ms). Ts was found to be shorter in Highly trained players compared to Elite players (42.33 ms vs. 197.39 ms).

### Meta-regression analysis

**FIG. 3:**
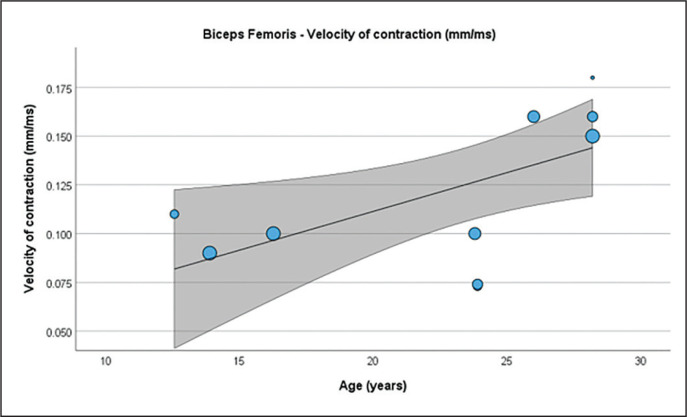
Results of the meta-regression analysis investigating the moderating effect of age on the velocity of contraction of the biceps femoris muscle in soccer players. Blue dots represent primary studies, solid lines denote meta-regression prediction lines, and the grey area indicates the 95% confidence intervals around the mean. Model: random-effects model; weights: random-effects; confidence intervals: estimated based on t-distribution.

**FIG. 4:**
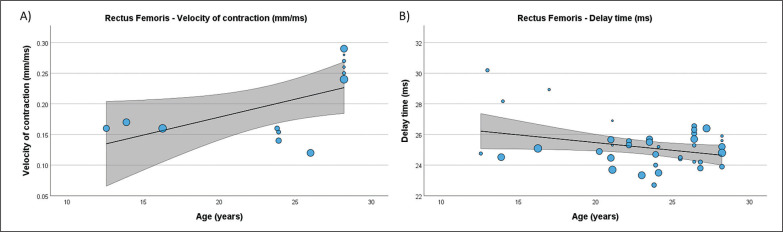
Results of the meta-regression analysis investigating the moderating effect of age on the velocity of contraction (A), and delay time (B), of the rectus femoris muscle in soccer players. Blue dots represent primary studies, solid lines denote meta-regression prediction lines, and the grey area indicates the 95% confidence intervals around the mean. Model: random-effects model; weights: random-effects; confidence intervals: estimated based on t-distribution.

**FIG. 5:**
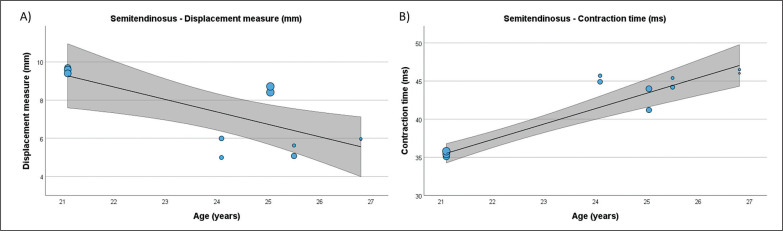
Results of the meta-regression analysis investigating the moderating effect of age on the displacement measure (A), and contraction time (B), of the semitendinosus muscle in soccer players. Blue dots represent primary studies, solid lines denote meta-regression prediction lines, and the grey area indicates the 95% confidence intervals around the mean. Model: random-effects model; weights: random-effects; confidence intervals: estimated based on t-distribution.

**FIG. 6:**
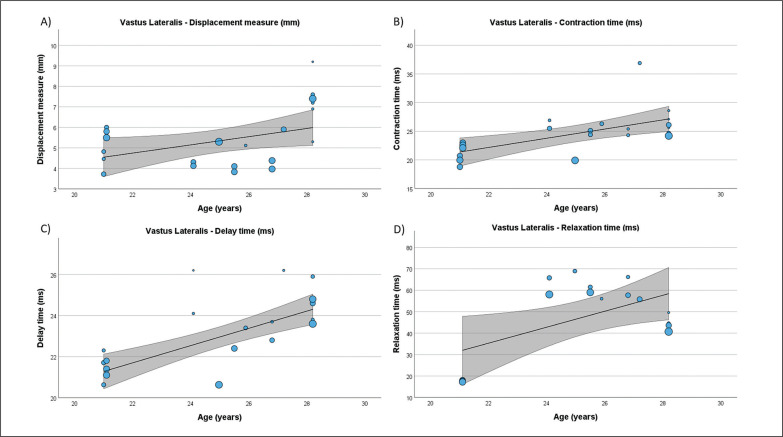
Results of the meta-regression analysis investigating the moderating effect of age on the displacement measure (A), contraction time (B), delay time (C), and relaxation time (D), of the vastus lateralis muscle in soccer players. Blue dots represent primary studies, solid lines denote meta-regression prediction lines, and the grey area indicates the 95% confidence intervals around the mean. Model: random-effects model; weights: random-effects; confidence intervals: estimated based on t-distribution.

**FIG. 7:**
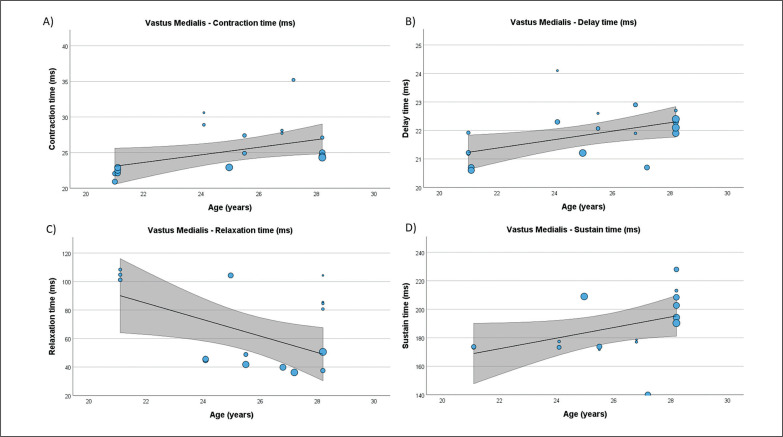
Results of the meta-regression analysis investigating the moderating effect of age on the contraction time (A), delay time (B), relaxation time (C), and sustain time (D), of the vastus medialis muscle in soccer players. Blue dots represent primary studies, solid lines denote meta-regression prediction lines, and the grey area indicates the 95% confidence intervals around the mean. Model: random-effects model; weights: random-effects; confidence intervals: estimated based on t-distribution.

Table 4 presents the results of meta-regression analysis, investigating whether the TMG-derived parameters in lower limb muscles were moderated by age of soccer players. Results demonstrate, that age moderated BF Vc (Fig. 3), RF Td and Vc (Fig.4), ST Dm and Ts (Fig. 5), VL Dm, Tc, Td and Tr (Fig. 6), and VM Tc, Td and Tr (Fig. 7), respectively.

**TABLE 4:** Results of the meta-regression analysis showing the associations between age and TMG-derived parameters in lower limb muscles of soccer players.

	Coefficient	Standard error	t value (n)	P value	95% lower CI	95% upper CI
**Biceps femoris**						
Dm (mm)	-0.04	0.07	-.56 (43)	.576	-0.19	0.10
Tc (ms)	-0.35	0.22	-1.60 (43)	.117	-0.80	0.09
Td (ms)	-0.03	0.06	-.57 (38)	.575	-0.16	0.09
Tr (ms)	1.61	2.23	.72 (25)	.477	-3.00	6.21
Ts (ms)	5.78	3.80	1.52 (25)	.142	-2.09	13.65
Vc (mm/ms)	0.004	0.002	2.62 (13)	.024	0.001	0.007

**Rectus femoris**						
Dm (mm)	0.07	0.06	1.29 (47)	.204	-0.04	0.18
Tc (ms)	-0.07	0.15	-.48 (47)	.635	-0.37	0.23
Td (ms)	-0.10	0.05	-2.05 (40)	.047	-0.20	0.00
Tr (ms)	1.94	2.04	.95 (24)	.353	-2.30	6.18
Ts (ms)	1.58	2.67	.59 (24)	.560	-3.95	7.11
Vc (mm/ms)	0.006	0.003	2.28 (13)	.043	0.00	0.012

**Semitendinosus**						
Dm (mm)	-0.65	0.20	-3.21 (11)	.011	-1.11	-0.19
Tc (ms)	2.02	0.25	8.15 (11)	< .001	1.46	2.58

**Vastus lateralis**						
Dm (mm)	0.20	0.10	2.10 (21)	.049	0.001	0.40
Tc (ms)	0.80	0.24	3.32 (21)	.004	0.30	1.31
Td (ms)	0.42	0.08	5.15 (21)	< .001	0.25	0.59
Tr (ms)	3.73	1.51	2.46 (18)	.026	0.52	6.94
Ts (ms)	3.79	2.85	1.33 (18)	.202	-2.25	9.82

**Vastus medialis**						
Dm (mm)	0.12	0.10	1.23 (20)	.234	-0.09	0.33
Tc (ms)	0.53	0.24	2.25 (20)	.037	0.04	1.03
Td (ms)	0.15	0.06	2.55 (20)	.020	0.03	0.27
Tr (ms)	-5.78	2.39	-2.41 (17)	.029	-10.88	-0.68
Ts (ms)	3.72	1.87	1.99 (17)	.066	-0.27	7.71

CI – confidence interval; n – number of mean values included in analysis; Dm – Displacement measure; Tc – Contraction time; Td – Delay time; Tr – Relaxation time; Ts – Sustain time; Vc – Velocity of contraction

## DISCUSSION

The present SLR with meta-analysis aimed to comprehensively analyse studies utilizing TMG to assess lower extremity muscles in soccer players. The initial search identified 210 reports, of which 25 articles from 22 studies involving 1224 participants were eligible for further analysis. The analysis considered various muscles, with the BF, RF, VL, VM, ST, GL, and GM being investigated. Significant variations were observed in TMG parameters across different muscles, age categories, and levels of play. For instance, BF exhibited significant variation in Dm, Tc, and Td across age categories, showing the smallest Dm and shortest Tc and Td in the U21 category. Differences in TMG parameters were also noted between elite, highly trained, and trained players, where BF Vc was found to be greater in Elite players compared to Highly trained players (0.13 mm/ms vs. 0.10 mm/ms), with a similar pattern observed for BF and VM Tc. Finally, the meta-regression analysis indicated that age moderated several TMG-derived parameters in lower limb muscles including BF Vc, RF Td and Vc, ST Dm and Ts, VL Dm, Tc, Td and Tr, and VM Tc, Td and Tr, respectively.

Significant variation between investigated muscles was found for all parameters of interest, including Tc, Dm, Td, Tr, Ts and Vc. For example, results showed that the shortest Tc was found in GL muscle (20.88 ms), followed by GM (22.09 ms), VL (24.56 ms), VM (25.34 ms), BF (28.74 ms), RF (29.32 ms), and ST (41.44 ms), respectively. Although, this SLR failed to quantify differences between female and male soccer players due lack of studies conducted on female athletes (N = 1), the current findings were similar with a recent cross-sectional study obtained in elite female players [4]. Later study showed that Tc was shorter in quadriceps muscles (VL = 21.2 ms, VM = 23.1 ms) compared to BF (29.2 ms) [4]. This, so called functional asymmetry in neuromuscular function between anterior and posterior thigh muscles, known as hamstring to quadriceps (H/Q) ratio has been observed in various populations [54–57]. Although, different methods exist, the H/Q ratio is mostly assessed utilizing dynamometers for force measurements [56, 57]. These asymmetries predominantly favour the quadriceps muscle, with the conventional H/Q ratio reference value is reported to be around 0.6 [56, 58]. In recent decades, sports scientists focused on investigating the H/Q ratio and its implication in predicting an ACL and hamstring injuries, albeit with limited evidence [59]. A recent literature review on this topic concluded that H/Q torque ratio has limited value in predicting ACL and hamstring injuries. Monitoring strength imbalances along, with other modifiable factors, through the entire competitive season may offer a better understanding of the association between H/Q ratio and injury occurrence is suggested [59]. The H/Q Tc ratio, calculated as an index of the average Tc of quadriceps muscles (VL, VM, RF) divided by the average Tc of BF and ST, was 0.75 in the current SLR. This is similar to results reported in a recent study for both the dominant (0.74) and non-dominant knee (0.78) of male amateur soccer players [60], however, lower than those observed in elite female soccer players (dominant knee = 0.80; non-dominant knee = 0.81) [51]. Thus, functional symmetry for the knee joint muscles can be captured utilizing TMG, however future studies investigating their implication for physical performance enhancement, injury prediction and prevention are warranted.

The neuromuscular function has been shown to be affected by many factors including an individual’s physical activity level [2, 61], age [62–65], sex [66], sport played [67] and level of play within a same sport [64, 68]. Thus, muscle contractile properties of male soccer players in general differ from those reported in older individuals diagnosed with chronic knee osteoarthritis [7], habitually inactive healthy adults [69], recreationally active adults [20] or solely power trained athletes [25]. For example, BF Tc observed in male soccer players from current study (28.74 ms), was found to be slightly shorter then Tc of elite female soccer players (29.3 ms) [4], highly trained amateur road cyclists (42.5 ms) [70], or Tier 2/Trained male soccer players (41.9 ms). However, it is twice longer then Tc in power-trained athletes (14.3 ms) [25]. Several studies have demonstrated training-specific changes in TMG-derived variables following exercise intervention [6, 20, 71–73]. For instance, Loturco and colleagues observed that an eight-week soccer-specific training, combined with endurance and strength-power exercises in elite soccer players, improved jumping ability alongside a reduction in Tc, Td and Dm of the BF [71]. Similar outcomes were reported after an eight-week of plyometric training in recreationally trained athletes [20]. Zubac and colleagues [20] reported a 12% increase in jumping height, accompanied by a reduction in VL Tc, BF Tc, tibialis anterior and GL Tc by 8.7%, 26.7%, 32.9% and 25.8%, respectively. Additionally, reductions of BF Dm (-26.5%), GM Dm (-14.9%) and GL Dm (-31.5) were observed. Dahmane and colleagues [74] found a high and positive correlation coefficient (r = 0.93) between TMG-derived Tc and histo-chemically determined percentage of MHC-I (i.e., slow twitch muscle fiber), suggesting that shorter Tc may indicate muscles with a higher percentage of fast twitch muscle fibers [14, 15]. Therefore, shorter Tc in power athletes compared to soccer players in general, could most likely be due to training specificity [75–77], resulting in a greater proportion of fast-twitch fibers i.e., MHC IIa and IIx [75] in measured muscle. Fast-twitch muscle fibers tend to shorten faster due to higher myosin ATPase activity and thus can generate more force and power [25, 78].

The TMG-derived Vc, calculated from the ratio between maximal radial displacement and the sum of contraction time and delay time, is an intriguing variable proposed for evaluating athlete neuromuscular function [71]. The current SLR found that BF Vc is greater in elite players compared to highly trained players (0.13 mm/ms vs. 0.10 mm/ms), with a similar pattern observed for RF Vc (Elite players = 0.21 mm/ms vs. Highly trained players = 0.16 mm/ms). While contraction velocity was shown to decrease with age linearly [79], it can be enhanced with physical training [80]. Thus, it can be concluded that Elite soccer players may counteract age-related negative changes of muscle contractile properties with a well-designed training regime [20, 61, 73]. These results also suggest that Vc is a sensitive marker for monitoring training-induced specific changes in muscle function and might serve as a valuable marker for talent identification, distinguishing between different levels of play in soccer.

Along with ACL tear, hamstring strain injuries are one of the most frequent occurring and reoccurring muscle injuries in team sports, including soccer [81, 82]. For example, BF was shown to be one of the most injured muscles in the hamstring muscles group, constituting 24% of all injuries in professional male soccer players [82]. The first study, employing TMG to differentiate between hamstring-injured and non-injured muscles, revealed a robust predictive capability of Tc parameter in functionally and non-invasively distinguishing between injured and non-injured BF with 98% accuracy [13]. Later study suggests that TMG can serve as a supplementary screening test in diagnosing BF injuries. The most recent study investigated whether muscle fibre typology might be the risk factor for hamstring strain injury [83]. Authors found that soccer players with fast fibre typology had a 5.3-fold greater risk of sustaining hamstring strain injury compared to players with pre-dominantly slow fibre typology. Age-related trends observed in the present study through meta-regression analysis for both BF and ST further emphasize the importance of considering age in injury prediction models. Epidemiological studies conducted among Australian football players [84] revealed that athletes aged 25 years and older exhibited a higher incidence of hamstring injuries (19.2%) compared to those aged 20 years and younger (6.9%). Another investigation including a similar population demonstrated an increased risk of hamstring injury for athletes aged 23 years and above [85]. Moreover, independent assessments, irrespective of previous injuries, indicated a 1.3-fold rise in the risk of hamstring injury for each additional year of age [86]. Hence, the results of the present SLR with meta-analysis could offer robust initial evidence that muscle contractile properties obtained through TMG might function as an indicator of an elevated risk of experiencing hamstring strain injuries. Nevertheless, further investigations are essential to delve into this matter, employing TMG alongside established methods for assessing muscle fiber typology among soccer players.

Some limitations of this SLR need acknowledgement and discussion. While the author generally adhered to PRISMA guidelines, certain exceptions were made that could limit the rigor of the review. Due to limited TMG-derived data reported for various age groups, female soccer players, different playing positions, and the definition of the dominant and non-dominant leg, the current SLR failed to provide a quantitative summary on these questions. Conversely, this may be considered a strength, as gaps in literature were properly identified, and new prospective studies investigating these questions with improved reporting on players’ characteristics are warranted.

## CONCLUSIONS

In conclusion, the current SLR with meta-analysis illuminated the multifaceted applications of TMG in assessing lower extremity muscles in soccer players. Beyond evaluating muscle contractile properties in various superficial muscles of the lower limbs in soccer players, TMG-derived parameters may serve as potentially valuable markers in identifying neuromuscular risk factors for ACL injuries and predicting hamstring-related injuries. The integration of age-related considerations and understanding of muscle typologies further refines the potential of TMG as a predictive tool in sports medicine, offering personalized insights for injury prevention, guiding rehabilitation strategies and potentially identifying talents in soccer.

## Supplementary Material

Establishing reference values for tensiomyography-derived parameters in soccer players: insights from a systematic review, meta-analysis and meta-regression

## Data Availability

Data will be provided upon the reasonable request to corresponding author.
