# Supplementary material for: Establishing reference values for tensiomyography-derived parameters in soccer players: insights from a systematic review, meta-analysis and meta-regression
Source: Biol Sport. 2024 Jul 31;42(1):171–92. doi: 10.5114/biolsport.2025.139853 (PMC11694203; doi:10.5114/biolsport.2025.139853)
Supplement: Establishing reference values for tensiomyography-derived parameters in soccer players: insights from a systematic review, meta-analysis and meta-regression [file JBS-42-54166-s1.pdf]

SUPPLEMENTARY MATERIALS

SUPPLEMENTARY TABLE 1. The methodological quality of included studies assessed by the quality assessment tool for observational cohort and cross-sectional studies.

	Criteria 1	Criteria 2	Criteria 3	Criteria 4	Criteria 5	Criteria 6	Criteria 7	Criteria 8	Criteria 9	Criteria 10	Criteria 11	Criteria 12	Criteria 13	Criteria 14	INDIVIDUAL STUDY REPORT		
Reference															YES	NO	NA
García-Manso et al. 2011	YES	NO	YES	YES	NO	NO	NO	NA	NO	NO	YES	NA	NA	NA	4	6	4
Rey et al. 2012	YES	YES	YES	YES	NO	YES	NO	NA	NO	NO	YES	NA	NA	NA	6	4	4
Rey et al. 2012	YES	YES	YES	YES	NO	YES	YES	NA	YES	NO	YES	NA	NA	NA	8	2	4
Rusu et al. 2013	YES	YES	YES	YES	NO	YES	NO	NA	NO	NO	YES	NA	NA	NA	6	4	4
Gil et al. 2015	YES	NO	YES	YES	YES	NO	NO	NA	NO	NO	YES	NA	NA	NA	5	5	4
Alentorn-Geli et al. 2015	YES	NO	YES	YES	YES	NO	NO	NA	NO	NO	YES	NA	NA	NA	5	5	4
Alvarez-Diaz et al. 2014	YES	NO	YES	YES	YES	NO	NO	NA	NO	NO	YES	NA	NA	NA	5	5	4
Alentorn-Geli et al. 2015	YES	NO	YES	YES	YES	NO	NO	NA	NO	NO	YES	NA	NA	NA	5	5	4
García-García et al. 2016	YES	YES	YES	YES	NO	YES	NO	NA	NO	YES	YES	NA	NA	NA	7	3	4
Alvarez-Diaz et al. 2016	YES	NO	YES	YES	YES	NO	NO	NA	NO	NO	YES	NA	NA	NA	5	5	4
Loturco et al. 2016	YES	YES	YES	YES	NO	YES	NO	NA	NO	NO	YES	NA	NA	NA	6	4	4
Alvarez-Diaz et al. 2016	YES	NO	YES	YES	YES	NO	NO	NA	NO	NO	YES	NA	NA	NA	5	5	4
García-García et al. 2017	YES	YES	YES	YES	NO	YES	NO	NA	NO	NO	YES	NA	NA	NA	6	4	4
López-Fernández et al. 2018	YES	NO	YES	YES	NO	NO	NO	NA	NO	NO	YES	NA	NA	NA	4	6	4
Loturco et al. 2018	YES	YES	YES	YES	NO	YES	NO	NA	NO	NO	YES	NA	NA	NA	6	4	4
Sánchez-Sánchez et al. 2019	YES	YES	YES	YES	NO	YES	NO	NA	NO	NO	YES	NA	NA	NA	6	4	4
Calderón-Pellegrino et al. 2020	YES	YES	YES	YES	NO	YES	NO	NA	NO	NO	YES	NA	NA	NA	6	4	4
Rey et al. 2020	YES	YES	YES	YES	NO	YES	YES	NA	YES	YES	YES	NA	NA	NA	9	1	4
Redd et al. 2021	YES	YES	YES	YES	NO	YES	NO	NA	NO	NO	YES	NA	NA	NA	6	4	4
Beato et al. 2021	YES	NO	YES	YES	NO	NO	NO	NA	NO	NO	YES	NA	NA	NA	4	6	4
Paravlic et al. 2022	YES	YES	YES	YES	YES	YES	YES	NA	YES	YES	YES	NA	NA	NA	10	0	4
Fernández-Baeza et al. 2022	YES	YES	YES	YES	NO	YES	YES	NA	YES	YES	YES	NA	NA	NA	9	1	4
Paravlic et al. 2022	YES	YES	YES	YES	YES	YES	YES	NA	YES	NO	YES	NA	NA	NA	9	1	4
Pajović et al. 2023	YES	YES	YES	YES	NO	YES	YES	NA	YES	NO	YES	NA	NA	NA	8	2	4
Padrón-Cabo et al. 2023	YES	YES	YES	YES	NO	YES	YES	NA	YES	NO	YES	NA	NA	NA	8	2	4

NA – not applicable; Criteria 1 – Was the research question or objective in this paper clearly stated?; Criteria 2 – Was the study population clearly specified and defined?; Criteria 3 – Was the participation rate of eligible persons at least 50%?; Criteria 4 – Were all the subjects selected or recruited from the same or similar populations (including the same time period)? Were inclusion and exclusion criteria for being in the study prespecified and applied uniformly to all participants?; Criteria 5 – Was a sample size justification, power description, or variance and effect estimates provided?; Criteria 6 – For the analyses in this paper, were the exposure(s) of interest measured prior to the outcome(s) being measured?; Criteria 7 – Was the timeframe sufficient so that one could reasonably expect to see an association between exposure and outcome if it existed?; "Criteria 8 – For exposures that can vary in amount or level, did the study examine different levels of the exposure as related to the outcome (e.g., categories of exposure, or exposure measured as continuous variable)?; Criteria 9 – Were the exposure measures (independent variables) clearly defined, valid, reliable, and implemented consistently across all study participants?; Criteria 10 – Was the exposure(s) assessed more than once over time?; Criteria 11 – Were the outcome measures (dependent variables) clearly defined, valid, reliable, and implemented consistently across all study participants?; Criteria 12 – Were the outcome assessors blinded to the exposure status of participants?; Criteria 13 – Was loss to follow-up after baseline 20% or less?; Criteria 14 – Were key potential confounding variables measured and adjusted statistically for their impact on the relationship between exposure(s) and outcome(s)?

SUPPLEMENTARY TABLE 2. Tensiomyography-derived parameters in football players across different levels of play.

			Mean value	SE	95% CI		95% PI		
TMG parameter	Tier classification / Level of play	n			Lower	Upper	Lower	Upper	Q value (p value)
				BICEPS FEMORIS					
Dm	Tier 2 Trained/Developmental	32	7.71	0.19	6.90	8.53	5.30	10.13	
	Tier 3 Highly Trained	364	5.26	0.30	4.61	5.90	3.11	7.40	
	Tier 4 Elite	675	4.97	0.42	4.10	5.84	0.52	9.43	
	Overall	1071	5.26	0.30	4.66	5.86	1.46	9.06	31.13 (< 0.001)
Tc	Tier 2 Trained/Developmental	32	41.86	0.59	39.31	44.42	34.31	49.42	
	Tier 3 Highly Trained	364	29.39	1.51	26.09	32.68	18.03	40.75	
	Tier 4 Elite	675	27.23	1.08	25.02	29.44	16.47	37.99	
	Overall	1071	28.74	0.96	26.79	30.68	17.14	40.33	33.77 (< 0.001)
Td	Tier 2 Trained/Developmental	32	24.66	0.16	23.96	25.36	22.59	26.72	
	Tier 3 Highly Trained	364	23.27	0.52	22.12	24.41	19.10	27.43	
	Tier 4 Elite	562	23.39	0.35	22.67	24.11	20.35	26.44	
	Overall	958	23.44	0.28	22.88	24.00	20.24	26.65	11.05 (0.004)
Tr	Tier 2 Trained/Developmental	32	62.57	0.58	55.19	69.95	.	.	
	Tier 3 Highly Trained	135	60.08	3.67	48.41	71.75	43.46	76.69	
	Tier 4 Elite	492	56.24	6.41	42.77	69.71	-3.99	116.46	
	Overall	659	57.62	4.97	47.37	67.88	5.59	109.66	0.38 (0.826)
Ts	Tier 2 Trained/Developmental	32	175.82	3.51	131.28	220.35	.	.	
	Tier 3 Highly Trained	135	194.64	9.17	165.44	223.84	115.32	273.97	
	Tier 4 Elite	492	194.21	12.13	168.73	219.69	84.18	304.24	
	Overall	659	193.20	9.24	174.12	212.28	100.51	285.90	2.95 (0.229)
Vc	Tier 3 Highly Trained	121	0.10	0.01	0.08	0.12	-0.01	0.21	
	Tier 4 Elite	122	0.13	0.01	0.11	0.16	0.04	0.23	
	Overall	243	0.13	0.01	0.10	0.15	0.04	0.21	6.96 (0.008)
				RECTUS FEMORIS					
Dm	Tier 2 Trained/Developmental	123	8.46	0.40	7.54	9.37	5.72	11.19	
	Tier 3 Highly Trained	364	9.5	0.31	8.83	10.17	7.40	11.60	
	Tier 4 Elite	355	8.77	0.44	7.85	9.68	4.47	13.06	
	Overall	842	8.89	0.26	8.37	9.42	5.58	12.21	4.66 (0.097)
Tc	Tier 2 Trained/Developmental	123	27	1.20	24.30	29.71	18.77	35.23	
	Tier 3 Highly Trained	364	32.33	1.69	28.64	36.02	18.84	45.83	
	Tier 4 Elite	355	28.6	0.69	27.17	30.04	21.72	35.49	
	Overall	842	29.32	0.68	27.95	30.69	20.30	38.34	6.98 (0.030)
Td	Tier 2 Trained/Developmental	63	24.784	0.37	23.83	25.74	22.30	27.27	
	Tier 3 Highly Trained	364	26.047	0.54	24.87	27.22	21.90	30.20	
	Tier 4 Elite	296	24.792	0.22	24.33	25.25	22.95	26.64	
	Overall	723	25.15	0.22	24.70	25.60	22.63	27.67	4.52 (0.104)
Tr	Tier 2 Trained/Developmental	32	95.23	5.60	24.11	166.36	.	.	
	Tier 3 Highly Trained	135	46.72	8.52	19.60	73.84	-32.35	125.79	
	Tier 4 Elite	226	73.6	5.31	62.39	84.80	30.09	117.10	
	Overall	393	70.18	4.93	59.98	80.38	24.11	116.26	12.69 (0.002)
Ts	Tier 2 Trained/Developmental	32	145.85	0.60	138.24	153.47	.	.	
	Tier 3 Highly Trained	135	88.26	14.15	43.23	133.29	-45.89	222.41	
	Tier 4 Elite	226	119.23	6.73	105.03	133.43	60.99	177.47	
	Overall	393	115.36	6.24	102.46	128.27	54.65	176.08	8.51 (0.014)

SUPPLEMENTARY TABLE 2. Continue.

			Mean value	SE	95% CI		95% PI		
TMG parameter	Tier classification / Level of play	n			Lower	Upper	Lower	Upper	Q value (p value)
Vc	Tier 3 Highly Trained	121	0.16	0.00	0.15	0.18	0.12	0.20	
	Tier 4 Elite	122	0.21	0.02	0.17	0.26	0.06	0.37	
	Overall	243	0.199	0.02	0.16	0.24	0.07	0.33	5.18 (0.023)
				VASTUS LATERALIS					
Dm	Tier 2 Trained/Developmental	31	4.29	0.34	2.82	5.75	-2.84	11.42	
	Tier 3 Highly Trained	147	5.72	0.12	5.38	6.05	5.33	6.10	
	Tier 4 Elite	413	5.47	0.46	4.47	6.46	2.02	8.92	
	Overall	591	5.32	0.29	4.72	5.93	2.74	7.91	13.34 (0.001)
Tc	Tier 2 Trained/Developmental	31	19.77	0.55	17.42	22.13	7.98	31.57	
	Tier 3 Highly Trained	147	26.14	2.77	18.45	33.83	4.73	47.56	
	Tier 4 Elite	413	24.94	0.58	23.67	26.21	20.45	29.43	
	Overall	591	24.56	0.84	22.82	26.31	16.55	32.58	42.28 (< 0.001)
Td	Tier 2 Trained/Developmental	31	21.47	0.48	19.40	23.54	11.82	31.12	
	Tier 3 Highly Trained	147	22.66	0.91	20.13	25.19	15.92	29.40	
	Tier 4 Elite	413	23.6	0.41	22.71	24.49	20.44	26.77	
	Overall	591	23.07	0.37	22.30	23.83	19.73	26.40	11.19 (0.004)
Tr	Tier 3 Highly Trained	147	31.99	9.28	6.22	57.75	-37.78	101.76	
	Tier 4 Elite	413	55.47	2.81	49.34	61.60	35.42	75.53	
	Overall	560	47.931	4.14	39.19	56.68	10.84	85.02	5.84 (0.016)
Ts	Tier 3 Highly Trained	147	69.05	14.30	29.36	108.74	-40.82	178.92	
	Tier 4 Elite	413	98.68	7.11	83.19	114.18	43.87	153.50	
	Overall	560	89.61	7.05	74.73	104.50	26.61	152.62	3.32 (0.068)
Vc	Tier 4 Elite	46	0.22	0.01	0.20	0.25	0.15	0.29	
				VASTUS MEDIALIS					
Dm	Tier 2 Trained/Developmental	31	7.02	0.29	5.76	8.28	1.21	12.83	
	Tier 3 Highly Trained	135	7.94	0.15	7.45	8.43	7.09	8.80	
	Tier 4 Elite	413	7.26	0.44	6.30	8.22	3.82	10.70	
	Overall	579	7.36	0.29	6.75	7.98	4.75	9.98	7.63 (0.002)
Tc	Tier 2 Trained/Developmental	31	21.36	0.39	19.67	23.06	13.29	29.44	
	Tier 3 Highly Trained	135	25.6	3.15	15.56	35.64	-4.41	55.61	
	Tier 4 Elite	413	25.7	0.57	24.46	26.94	21.73	29.66	
	Overall	579	25.34	0.77	23.73	26.94	18.38	32.30	31.94 (< 0.001)
Td	Tier 2 Trained/Developmental	31	21.43	0.24	20.41	22.44	17.65	25.20	
	Tier 3 Highly Trained	135	20.66	0.03	20.58	20.75	20.55	20.78	
	Tier 4 Elite	413	22.29	0.18	21.90	22.69	21.00	23.58	
	Overall	579	21.834	0.20	21.42	22.25	20.06	23.61	62.46 (< 0.001)
Tr	Tier 3 Highly Trained	135	86.78	17.49	31.11	142.44	-80.27	253.82	
	Tier 4 Elite	413	57.08	6.50	42.91	71.25	8.36	105.81	
	Overall	548	65.15	6.96	50.40	79.91	5.13	125.18	2.46 (0.117)
Ts	Tier 3 Highly Trained	135	164.886	8.39	138.19	191.58	86.46	243.31	
	Tier 4 Elite	413	193.099	5.14	181.91	204.29	152.75	233.45	
	Overall	548	186.165	5.21	175.13	197.20	140.15	232.18	8.12 (0.004)

CI – confidence interval; n – number of players included in the analysis; SE – standard error; PI – prediction interval; Dm – Displacement measure; Tc – Contraction time; Td – Delay time; Tr – Relaxation time; Ts – Sustain time; Vc – Velocity of contraction.

SUPPLEMENTARY TABLE 3. Tensiomyography-derived parameters in football players across age categories.

			Mean value	SE	95% CI		95% PI		
TMG parameter	Age category	n			Lower	Upper	Lower	Upper	Q value (p value)
				BICEPS FEMORIS					
Dm	Senior	905	5.18	0.34	4.50	5.87	1.16	9.21	
	U14 (12 to 13)	39	5.99	0.08	4.94	7.04	.	.	
	U16 (14 to 15)	57	6.25	1.48	-12.61	25.11	.	.	
	U19 (16 to 18)	87	6.12	0.57	-1.09	13.33	.	.	
	U21 (19 to 21)	15	3.15	0.21	.	.	.	.	
	Overall	1103	5.26	0.30	4.66	5.86	1.46	9.06	72.75 (< 0.001)
Tc	Senior	905	27.92	1.02	25.85	29.99	16.72	39.12	
	U14 (12 to 13)	39	36.62	1.07	23.02	50.22	.	.	
	U16 (14 to 15)	57	32.83	1.53	13.42	52.24	.	.	
	U19 (16 to 18)	87	34.60	3.05	-4.22	73.42	.	.	
	U21 (19 to 21)	15	22.17	0.93	.	.	.	.	
	Overall	1103	28.74	0.96	26.79	30.68	17.14	40.33	70.09 (< 0.001)
Td	Senior	792	23.33	0.29	22.73	23.92	20.30	26.36	
	U14 (12 to 13)	39	24.68	1.62	4.04	45.33	.	.	
	U16 (14 to 15)	57	24.03	1.95	-0.75	48.80	.	.	
	U19 (16 to 18)	87	24.14	1.82	1.04	47.24	.	.	
	U21 (19 to 21)	15	21.93	0.33	.	.	.	.	
	Overall	990	23.44	0.28	22.88	24.00	20.24	26.65	12.46 (0.014)
Tr	Senior	659	57.62	4.97	47.37	67.88	5.59	109.66	NA
Ts	Senior	659	193.20	9.24	174.12	212.28	100.51	285.90	NA
Vc	Senior	122	0.13	0.01	0.11	0.16	0.04	0.23	
	U14 (12 to 13)	18	0.11	0.01	.	.	.	.	
	U16 (14 to 15)	37	0.09	0.01	.	.	.	.	
	U19 (16 to 18)	66	0.10	0.01	.	.	.	.	
	Overall	243	0.13	0.01	0.10	0.15	0.04	0.21	12.55 (0.006)
				RECTUS FEMORIS					
Dm	Senior	616	9.07	0.32	8.42	9.73	5.43	12.72	
	U14 (12 to 13)	39	8.64	0.17	6.46	10.82	.	.	
	U16 (14 to 15)	57	9.99	0.57	2.71	17.28	.	.	
	U19 (16 to 18)	147	7.95	0.53	6.59	9.31	4.33	11.57	
	U21 (19 to 21)	15	7.59	0.50	.	.	.	.	
	Overall	874	8.89	0.26	8.37	9.42	5.58	12.21	13.35 (0.010)
Tc	Senior	616	28.85	0.58	27.67	30.03	22.02	35.69	
	U14 (12 to 13)	39	36.73	6.25	-42.68	116.14	.	.	
	U16 (14 to 15)	57	35.34	5.02	-28.39	99.07	.	.	
	U19 (16 to 18)	147	28.06	3.06	20.19	35.93	6.17	49.96	
	U21 (19 to 21)	15	28.44	1.14	.	.	.	.	
	Overall	874	29.32	0.68	27.95	30.69	20.30	38.34	3.46 (0.484)

SUPPLEMENTARY TABLE 3. Continue.

TMG parameter	Age category	n	Mean value	SE	95% CI Lower	95% CI Upper	95% PI Lower	95% PI Upper	Q value (p value)
Td	Senior	557	24.88	0.17	24.52	25.23	23.08	26.67	
	U14 (12 to 13)	39	27.48	2.72	-7.02	61.98	.	.	
	U16 (14 to 15)	57	26.28	1.82	3.10	49.45	.	.	
	U19 (16 to 18)	87	26.86	1.91	2.54	51.18	.	.	
	U21 (19 to 21)	15	24.89	0.47	.	.	.	.	
	Overall	755	25.15	0.22	24.70	25.60	22.63	27.67	2.529 (0.640)
Tr	Senior	393	70.18	4.93	59.98	80.38	24.11	116.26	NA
Ts	Senior	393	115.36	6.24	102.46	128.27	54.65	176.08	NA
Vc	Senior	122	0.21	0.02	0.17	0.26	0.06	0.37	
	U14 (12 to 13)	18	0.16	0.01	.	.	.	.	
	U16 (14 to 15)	37	0.17	0.01	.	.	.	.	
	U19 (16 to 18)	66	0.16	0.01	.	.	.	.	
	Overall	243	0.20	0.02	0.16	0.24	0.07	0.33	6.65 (0.084)
**VASTUS LATERALIS**									
Dm	Senior	591	5.33	0.29	4.72	5.93	2.74	7.91	NA
Tc	Senior	591	24.56	0.84	22.82	26.31	16.55	32.58	NA
Td	Senior	591	23.07	0.37	22.30	23.83	19.73	26.40	NA
Tr	Senior	560	47.93	4.14	39.19	56.68	10.84	85.02	NA
Ts	Senior	560	89.61	7.05	74.73	104.50	26.61	152.62	NA
Vc	Senior	33	0.22	0.01	0.20	0.25	0.15	0.29	NA
**VASTUS MEDIALIS**									
Dm	Senior	579	7.36	0.29	6.75	7.98	4.75	9.98	NA
Tc	Senior	579	25.34	0.77	23.73	26.94	18.38	32.30	NA
Td	Senior	579	21.83	0.20	21.42	22.25	20.06	23.61	NA
Tr	Senior	548	65.15	6.96	50.40	79.91	5.13	125.18	NA
Ts	Senior	548	186.17	5.21	175.13	197.20	140.15	232.18	NA

CI – confidence interval; n – number of players included in the analysis; SE – standard error; PI – prediction interval; Dm – Displacement measure; Tc – Contraction time; Td – Delay time; Tr – Relaxation time; Ts – Sustain time; Vc – Velocity of contraction.
